# An Easy Method of Refining Gold

**Published:** 1899-03

**Authors:** A. D. Hooker

**Affiliations:** San Jose, Cal.


ARTICLE VI.

### AN EASY METHOD OF REFINING GOLD.

DR. A. D. HOOKER, SAN JOSE, CAL.

Since the introduction of crown and bridge work the busy dentist finds his gold drawer gradually filling up with scraps of gold ranging in quality from eighteen to twenty-four carats fine.

The work of refining gold by any of the ordinary processes is not only difficult for the average dentist, but it takes a great deal of time and skill.

The process of refining to which we now desire to call attention is very easy and simple. One which the student or office boy could work out with very little trouble. It is briefly as follows:

Gather up all of the old scraps and filings, carefully discarding all stray pieces of platinum which may be mixed with the gold. Take four parts of sheet copper to one part of gold scraps, melt all together in a crucible, or it may be done with the blow-pipe, with foot-blower attachment using a large piece of charcoal or asbestos cup to melt it. After the two metals have been perfectly melted and thoroughly mixed, cool off and place the mass on an anvil or swaging block and with a four-pound hammer reduce to a thin sheet. Then run it through the rolling-mill until the whole mass is as thin as tissue paper. Boil out in soap and water to remove any oil which may have gathered upon it during the process of rolling. Now cut the sheet or sheets of metal into narrow strips about one-fourth of an inch in width, and place them in an earthern vessel and set outside of the office window. Pour into the vessel containing the metal sufficient commercial nitric acid to attack and eat up the copper, which it will do very quickly if everything is working right. After letting it stand a short time to cool, the acid may be carefully poured out so as not to disturb the gold which will be found in the bottom of the bowl or vessel.

At first sight one would almost believe that this black, dirty-looking deposit was worthless, and that the gold had been ruined by the refining process. It is only necessary, however, to carefully wash and rinse with clean water to bring the gold plainly into view.

Gather up the fine dust, dry, melt and roll again into any thickness desired.

If every detail of the process has been well done the gold will be pure, twenty-four carats fine and as soft as lead.

In remelting and rolling clippings and scraps of twenty and twenty-two carat gold, which has been kept free from all other grades, we naturally expect to work well without any refining; but it does not always do so. It will sometimes crack under the hammer and act in a fractious and unbecoming manner under the roller.

To make this again ductile and pliable it will only be necessary to place it on a piece of charcoal (first making a cone-shaped depression in it.) And with the blow-pipe melt and boil it till very hot, and while it is still boiling throw on to the molten mass a small piece of crorosive sublimate followed by a little saltpeter. This will clean it up and make it again pliable.—*Pacific Medico-Dental Gazette.*

